# Dental Tissue-Derived Human Mesenchymal Stem Cells and Their Potential in Therapeutic Application

**DOI:** 10.1155/2020/8864572

**Published:** 2020-09-01

**Authors:** Lu Gan, Ying Liu, Dixin Cui, Yue Pan, Liwei Zheng, Mian Wan

**Affiliations:** ^1^State Key Laboratory of Oral Diseases and National Clinical Research Center for Oral Diseases and Department of Pediatric Dentistry, West China Hospital of Stomatology, Sichuan University, Chengdu, Sichuan 610041, China; ^2^State Key Laboratory of Oral Diseases and National Clinical Research Center for Oral Diseases and Department of Cariology and Endodontics, West China Hospital of Stomatology, Sichuan University, Chengdu, Sichuan 610041, China

## Abstract

Human mesenchymal stem cells (hMSCs) are multipotent cells, which exhibit plastic adherence, express specific cell surface marker spectrum, and have multi-lineage differentiation potential. These cells can be obtained from multiple tissues. Dental tissue-derived hMSCs (dental MSCs) possess the ability to give rise to mesodermal lineage (osteocytes, adipocytes, and chondrocytes), ectodermal lineage (neurocytes), and endodermal lineages (hepatocytes). Dental MSCs were first isolated from dental pulp of the extracted third molar and till now they have been purified from various dental tissues, including pulp tissue of permanent teeth and exfoliated deciduous teeth, apical papilla, periodontal ligament, gingiva, dental follicle, tooth germ, and alveolar bone. Dental MSCs are not only easily accessible but are also expandable *in vitro* with relative genomic stability for a long period of time. Moreover, dental MSCs have exhibited immunomodulatory properties by secreting cytokines. Easy accessibility, multi-lineage differentiation potential, and immunomodulatory effects make dental MSCs distinct from the other hMSCs and an effective tool in stem cell-based therapy. Several preclinical studies and clinical trials have been performed using dental MSCs in the treatment of multiple ailments, ranging from dental diseases to nondental diseases. The present review has summarized dental MSC sources, multi-lineage differentiation capacities, immunomodulatory features, its potential in the treatment of diseases, and its application in both preclinical studies and clinical trials. The regenerative therapeutic strategies in dental medicine have also been discussed.

## 1. Introduction

Human mesenchymal stem cells (hMSCs) are multipotent cells isolated from various tissues, including bone marrow, adipose tissue, umbilical cord, and dental tissue. These cells share similar properties: being plastic-adherent, expressing a specific cell surface marker spectrum (CD73+, CD90+, CD105+, CD34-, CD45-, CD11b-, CD14-, CD19-, CD79a-, and human leucocyte antigen-DR-), and possessing the ability to give rise to osteoblasts, chondrocytes, and adipocytes. hMSCs are highly accessible and expandable *in vitro* with genomic stability. Furthermore, these cells have the remarkable potential of multipotent differentiation, as they not only could differentiate into mesodermal lineages (adipocytes, osteocytes, and chondrocytes) but also could transdifferentiate into ectodermal lineages (neurocytes) and endodermal lineages (hepatocytes and pancreocytes). All these characteristics make them promising stem cell sources for regenerative therapy, but their clinical applications have been limited due to questionable safety issues, inconclusive quality control, unaccomplished clinical-grade production, and incomplete understanding of the mechanism regulating these hMSCs [1–4].

**Figure 1:**
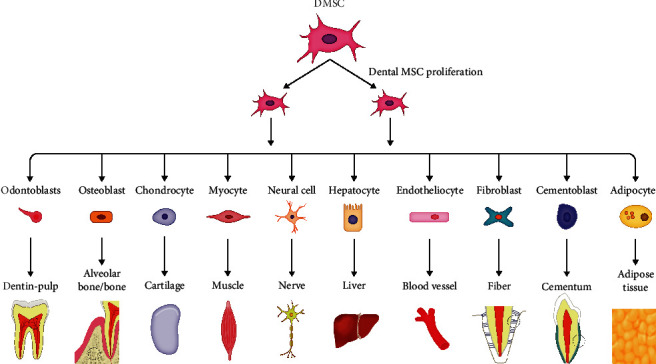
Multilineage differentiation capacity of dental MSCs.

To address this, scientists worldwide have been searching for safe, effective, and easily accessible stem cell sources with great differentiation potential for regenerative medicine. Dental MSCs, which show typical MSC features, have been found in various dental tissues, ranging from discarded extracted teeth to their attached tissues [5–7]. These cells are not only easily accessible but are also expandable with relative genomic stability for a long period of time. Notably, apart from mesodermal lineages, they have the ability to transdifferentiate into ectodermal and endodermal lineages [5, 8–12] (Figure 1). Moreover, dental MSCs exhibit immunomodulatory properties by secreting cytokines and immune receptors [13]. All these characteristics of dental MSCs make them distinct from other hMSCs, and they can be applied in stem cell-based therapy. Several preclinical studies and clinical trials were performed using dental MSCs in the treatment of dental diseases and nondental diseases like neurodegenerative diseases and autoimmune and orthopedic disorders [14–18].

The present review has summarized dental MSC sources, multi-lineage differentiation potential, immunomodulatory features, its potential in the treatment of diseases, and its application in both preclinical studies and clinical trials. The regenerative therapeutic strategies in dental medicine have also been discussed.

## 2. Dental Mesenchymal Stem Cells (Dental MSCs)

The existence of dental MSCs was suggested by the formation of tertiary dentin following dental caries or trauma. The first efficient population of dental MSCs was reported from the dental pulp tissue of an extracted third molar [19]. Later on, cells that possess characteristics of MSCs were isolated from the pulp tissue of exfoliated deciduous teeth [20], apical papilla [21], periodontal ligament [22], gingiva [23], dental follicle [24, 25], tooth germ [26], and alveolar bone [27]. These cell populations exhibit heterogeneity, i.e., distinct cell surface markers, proliferation rate, and differentiation potential, which has been reviewed by Zhou et al. [28], suggesting their diverse functions and applications in clinic.

### 2.1. Stem Cells from Dental Pulp

The first dental MSC population, human dental pulp stem cells (hDPSCs), was isolated from the dental pulp tissue of impacted third molars. These cells exhibit MSC properties, including high proliferation, multi-lineage differentiation potential, as well as immunomodulatory properties [19, 29]. Substantial studies have documented the odontogenic differentiation potential of hDPSCs, i.e., hDPSCs generated a dentin–pulp-like organoid with Matrigel *in vitro* and induced mineralized reparative dentin formation with hydroxyapatite (HA)/tricalcium phosphate (TCP) ceramic particles *in vivo* [19, 30–32]. Attributing to the origin of the neural crest, hDPSCs show remarkable neurogenic potential compared with human bone marrow stem cells (BMMSCs). The higher expression level of neurotrophins like nerve growth factor (NGF) and longer axons were detected in hDPSCs cultured with a microfluidic coculture system containing trigeminal neurons. Neurospheres were also generated by hDPSCs upon specific differentiation conditions [33–35]. The ability of hDPSCs to differentiate into endothelial cells and their angiogenic potential have also been reported, as hDPSCs were found to secrete vascular endothelial growth factors (VEGF) and generate visible blood vessels in three-dimensional- (3D-) printed HA constructs [36]. Their capabilities of neurogenic and angiogenic differentiation made a great contribution to the whole pulp regeneration. Implanted hDPSCs gave rise to 3D pulp tissue with vascular and nerve reconstruction in the empty root canal of traumatized permanent incisors [37]. hDPSCs could also differentiate into osteoblasts and further regenerate bone tissue, due to expressing several typical osteoblastic markers, such as alkaline phosphatase (ALP), osteopontin (OPN), and osteocalcin (OCN) [38]. Newly formed bone was found following the application of the bioengineered constructs of hDPSCs with poly-*ɛ*-caprolactone- (PCL-) biphasic calcium phosphate (BCP) scaffolds. hDPSCs could also differentiate into other cell lineages, such as adipocytes, chondroblasts, hepatocytes, and cardiomyocytes. The high plasticity of hDPSCs makes them an ideal stem cell source for stem cell-based therapy, which has been thoroughly reviewed by Mortada et al. [29].

Then, stem cells from the dental pulp tissue of exfoliated deciduous teeth were purified with a similar method for hDPSC isolation. Analogous to hDPSCs, cultured stem cells from exfoliated deciduous teeth (SHEDs) are capable of differentiating into various cell types, such as osteocytes, chondrocytes, adipocytes, odontoblasts, endothelial cells, and hepatocytes [20]. However, due to the developmental differences between deciduous and permanent teeth, SHEDs present different features from hDPSCs, for instance, a higher proliferative capability, more cell-population doublings, a sphere-like cluster formation, and a distinctive osteoinductive capacity [20]. For odontogenic differentiation potential, SHEDs are able to differentiate into odontoblasts and form dentin-like tissue or pulp-like tissue, instead of a complete dentin–pulp-like complex [20, 39]. When combined with collagen type I, hDPSCs formed the functional dental pulp tissue in the full-length root canal. The newly formed pulp tissue contained functional odontoblasts, which regenerated tubular dentin tissue [40]. Following neural inductive culture, SHEDs presented higher expression levels of neuronal and glial cell markers than hDPSCs, such as *β*-III-tubulin, tyrosine-hydroxylase (TH), microtubule-associated protein 2 (MAP2), and Nestin. Dopaminergic (DAergic) neurons could be produced by SHED-derived neurospheres in a DAergic induction system [41]. Additionally, SHEDs could act as neuroprotector agents to promote neural functional recovery through paracrine effects and inhibit glial scar formation after spinal cord contusion [42]. Conditioned media (CM) derived from SHEDs, containing various neurotrophic factors, enhanced peripheral sciatic nerve regeneration with axon regeneration and remyelination, which improved motor functions thus preventing muscle atrophy [43]. These multifaceted neural regeneration activities render SHEDs as an optimal cellular source to improve the injured nerve. For osteogenic potential, SHEDs induced new bone formation *in vivo* by recruiting host osteogenic cells, rather than differentiating into osteoblasts which happened *in vitro* [20]. Larger osteoids and more collagen fibers were formed by SHEDs with polylactic-coglycolic acid (PLGA) membrane transplantation as compared to DPSCs and BMMSCs [44].

In addition to striking multi-lineage differentiation potential, the immunomodulatory effects have been reported in MSCs from dental pulp tissue, which may function through correcting the underlying pathological immune responses. hDPSCs have been suggested to regulate local immune response by suppressing the expression of metalloproteinases (MMPs) including MMP3 and MMP13 and to inhibit acute rejection of allograft by releasing transforming growth factor-beta (TGF-*β*) [45]. Dai *et al.* found that SHEDs suppressed the CD4+ T cell-driven responses via inhibiting the proliferation of T lymphocytes and the upregulated ratio of Th1/Th2 by inducing the expansion of Treg cells [46]. Local injection of SHEDs increased the number of anti-inflammatory CD206+ M2 macrophages and altered the cytokine expression profiles in periodontal tissues with periodontitis [47].

### 2.2. Stem Cells from the Apical Papilla (SCAPs)

During tooth development, dental papilla derived from the ectomesenchyme ultimately converts into the dental pulp tissue and migrate to locations around the apex [48]. Root development and apical closure could still be observed in immature permanent teeth suffering from periapical periodontitis or abscess. These clinical phenomena suggested that a population of MSCs might reside in apical papilla. SCAPs were isolated from third molar root apical papilla, which contains fewer blood vessels and cells than dental pulp tissue [21, 48].

SCAPs have shown a greater potential to regenerate dentin than DPSCs, since they have higher proliferation with greater telomerase activity, suggesting that SHED is a cell source for odontoblasts responsible for the production of dentin [21]. Previous studies have confirmed that SCAPs are capable of differentiating into odontoblast-like cells and form a typical dentin-like structure on the surface of HA/TCP [21, 48]. Larger areas of mineralized nodules positive to Alizarin Red were formed by SCAPs with culture medium containing L-ascorbate-2-phosphate [48]. When SCAPs mixed with host cells, odontoblasts positive for dentin sialophosphoprotein (DSPP) and dentin matrix protein 1 (DMP1) and ectopic formation of vascularized pulp-like tissue were detected in mice molars [49]. A greater migration ability assessed by scratch assay enhanced their capacity for dentin regeneration by cell homing [21]. Considering their role in root development, SCAPs have been suggested to possess a significant potential for root regeneration. A functional bioroot with periodontal ligament tissue was generated in the alveolar socket of a minipig following transplantation of human SCAPs and periodontal ligament stem cells (PDLSCs). Additionally, SHEDs showed a PDL-related marker *in vitro* and exhibited greater mineralization capacity on account of higher expression levels of ALP, bone sialoprotein (BSP), and OCN expression compared to PDLSCs [50]. Therefore, SCAPs have been considered as a promising alternative source for periodontal tissue regeneration. Their potential for angiogenesis has also been confirmed in 3D-printed HA scaffolds. Derived from the cranial neural crest, SCAPs possess neurogenic differentiation potential similar to DPSCs and SHEDs. After transplantation of the human apical papilla tissue into the injured spinal cord in rats, improvements were observed in gait and glial reactivity [51]. Besides, SCAPs may be a potential immunotherapeutic tool for immunological diseases due to their low immunogenicity and capability of inhibiting T cell proliferation [52].

### 2.3. Periodontal Ligament Stem Cells (PDLSCs)

Periodontal ligament (PDL) is a soft connective tissue, which contains progenitor cells that maintain tissue homeostasis and regeneration of periodontal tissues [22, 53]. PDLSCs were isolated from the attached PDL of the extracted third molar with expression of two early MSC markers STRO-1 and CD146/MUC18 and higher levels of scleraxis, a tendon-specific transcription factor, compared to DPSCs [22].

The potential for the cementogenic/osteogenic differentiation of PDLSCs has been shown by the formation of calcified nodules and expression of ALP, matrix extracellular protein (MEPE), BSP, OCN, and TGF-*β* receptor I [22]. Human PDLSCs have been demonstrated to be a reliable source for the fabrication of 3D PDL tissues [54]. Typical cementum/PDL-like structures, including Sharpey's fiber-like tissue, were generated after the transplantation of human PDLSCs into the rat periodontal lesion sites [22]. PDLSCs also contribute to root regeneration. When combined with SCAPs, they generated a collagen fiber which anchored into the newly formed cementum on the surface of the HA/TCP carrier, and formed a functional root supporting a porcelain crown [21]. Extracellular vesicles (EVs) released by PDLSCs have also been reported to possess osteogenic properties and promote bone regeneration. Collagen membranes with PDLSC-EV transplantation showed osteoid formation with an osteoblast-like structure on the host native bone side and new bone irregularly arranged in the implant site of rats subjected to calvarial defects. Neural crest-derived PDLSCs spontaneously express neural protein markers as Nestin and growth associated protein-43 (GAP-43) upon xeno-free culture conditions [55]. In addition to previously demonstrated adipocytes and chondrogenic cells, several studies sequentially reported the differentiation potential of PDLSCs toward endothelial cells, cardiac myocytes, islet-like cells, and retinal ganglion-like cells [11]

Based on immunosuppression, low immunogenicity, and the ability to produce a vast array of cytokines, PDLSCs and their products have the potential to treat inflammatory disorders and autoimmune diseases. Ding *et al.* found the PDLSCs failed to express HLA-II DR and costimulatory molecules and possessed marked immunosuppression via PGE2-induced T-cell anergy [56].

### 2.4. Gingival-Derived Mesenchymal Stem Cells (GMSCs)

A population of progenitor cells or stem cells has been identified in the spinous layer of human gingiva, an easily accessible tissue from remnants or discarded tissues following routine dental procedures, namely GMSCs.

The osteogenic differentiation of GMSCs was determined by formed deposits with positive Alizarin Red S staining and upregulated expression of OCN *in vitro*, while their ability for osteogenic differentiation has not been observed *in vivo* [23]. However, recent evidence has shown that EVs derived from GMSCs exhibit significant osteogenic properties as revealing high expression levels of RUNX2 and BMP2/4 and abundant extracellular matrix (ECM) and nodules of new bone formation [57]. Transplantation of GMSCs formed connective-like tissues expressing collagen I, which is absent in DPSCs or PDLSCs [23]. Human fetal GMSCs have an ability for gingival differentiation automatically *in vivo* because they may contain more precursor cells to differentiate into gingival cells. After having been transplanted into the gingiva defects of rats, human GMSCs generated new tissue like normal gingiva [58]. GMSCs are capable of neurogenic differentiation since they are positive for glial fibrillary acidic protein (GFAP), neurofilament (NF-M), and *β*-tubulin III upon neural differentiation conditions [23]. GMSC spheroids have shown differentiation potential into both neuronal and Schwann-like cells with a 3D-collagen scaffold. And 3D bioprinted grafts with GMSCs formed nerve tissue with a normal size at the defect of rat facial nerves and showed higher therapeutic potential on facial palsy. These findings have demonstrated that GMSCs present promising potential for nerve regeneration and functional recovery [59]. Besides, they could also differentiate into adipocytes, chondrocytes and endothelial cells [23, 60].

Importantly, GMSCs have distinctive immunomodulatory functions, as they could suppress peripheral blood mononuclear cells (PBMCs) and upregulate IFN-*γ*-induced indoleamine 2,3-dioxygenase(IDO) and IL-10. Spheroid-derived GMSCs displayed the capability to enhance the secretion of several chemokines and cytokines and improved resistance to oxidative stress-induced apoptosis. They have been reported to attenuate chemotherapy-induced oral mucositis [61].

### 2.5. Dental Follicle Stem Cells (DFSCs)

The dental follicle (DF) is responsible for forming alveolar bone and the root-bone interface in tooth development; it is an ectomesenchymal tissue that contains progenitor cells (PCs) for periodontal ligament cells, cementoblasts, and osteoblasts [24, 25, 28]. These PCs were isolated from the dental follicle of the extracted third molars, characterized by expressed undifferentiated cell markers Notch-1 and Nestin, namely DFSCs [24, 25].

DFSCs express a higher level of insulin-like growth factors (IGF-2) compared to hMSCs and exhibit higher proliferation potential and colony-forming ability compared with SHEDs, DPSCs, and PDLSCs, suggesting their potential in regenerative medicine [24, 25, 62, 63]. Their superior osteogenic properties have been reported by several studies. DFSCs show higher expression levels of osteogenic-related markers such as RUNX2 and ALP compared to SHEDs and DPSCs [62]. Long-term culture of DFSCs with differentiation inductive medium have demonstrated that they have the potential to differentiate into osteoblasts expressing BSP and OCN and form calcium deposits [24, 25]. DFSCs are more immature than PDLSCs. There is less heterochromatin in the nucleus and fewer organelles and bundles of microfilaments in the cytoplasm of DFSCs than in the cytoplasm of PDLSCs on ultrastructural comparison [63]. The higher expression of DSPP in DFSCs has shown its preferable potential for odontogenic differentiation and dentin regeneration compared to PDLSCs. And they generated complete dentin including dentin, predentin, and calcospherites upon the induction of treated dentin matrix (TDM) [11]. The properties of periodontal differentiation of DFSCs have also been demonstrated. They are able to form fibrous membrane PDL-like structures or calcified nodules with bone- or cementum-like structures under *in vitro* conditions, suggesting their potential for periodontal differentiation [24, 25]. Upon *in vivo* transplantation, DFSCs derived from the apical end of human developing root could produce a cementum/PDL-like complex characterized by a thin layer of cementum-like tissues and PDL-like collagen fibers inserted perpendicularly into the newly formed cementum-like deposits [25]. These findings suggest that DFSCs are a promising alternative source for bioroot engineering.

Furthermore, the immunomodulatory effects of DFSCs also favor their therapeutic potential to treat autoimmune, inflammatory, and allergic diseases. Compared with SHEDs and DPSCs, DFSCs stimulated by IFN-*γ* remarkably increased the number of CD4+FOXP3+ Treg cells and suppressed the proliferation and apoptosis of peripheral blood mononuclear cells (PBMCs) [62, 64].

### 2.6. Tooth Germ Stem Cells (TGSCs)

In the bell stage, tooth germ consists of three components including enamel organ, dental mesenchymal cells (dental papilla or pulp), and dental follicle. The progenitor cell populations of dental mesenchymal cells, named TGSCs, have been isolated and identified from human third molar tooth germ [26]. The expression of DSPP has confirmed the odontogenic differentiation of TGSCs with the treatment of BMP2 and BMP7 [65].

The osteogenic differentiation capability of TGSCs has been demonstrated, as new bone formation was obtained in the pore area of the HA/TGSC implants. TGSCs have the potential to regenerate cartilage tissue, which is attributed to their chondrogenic differentiation ability upon induction. After TGSCs attached to 3D biological scaffolds, abundant hyaline cartilage-specific extracellular matrix (ECM) and collagen type II expression were found [66]. TGSCs were also able to differentiate into hepatocytes under hepatic induction. This was indicated by the expression of the liver-specific albumin gene, positive staining for albumin protein, and morphological change [26]. In rats with injured liver, transplantation of differentiated TGSCs could suppress the liver hydroxyproline content and reduce areas of damage, therefore suppressing liver fibrosis and steatonecrosis [26], suggesting that TGSCs are useful in cytotherapy for liver diseases.

### 2.7. Alveolar Bone-Derived Mesenchymal Stem Cells (ABMSCs)

BMMSCs have been isolated from various bone tissues such as the ilium by an invasive procedure, namely, marrow aspiration. Alternatively, collecting ABMSCs from alveolar bone during the course of dental surgery is providing a new isolation method with a few extra invasive interventions [27]. ABMSCs have favorable osteogenic differentiation potential comparable to BMMSCs but a weaker potential to differentiate into chondrocytes or adipocytes [27]. New bone has been detected in a rabbit critical-size mandibular bone defect model with transplants which consist of ABMSCs and *β*-TCP [67]. ABMSCs also have the potential for bone tissue regeneration, and their potential to reconstruct alveolar bone will contribute to improving periodontal defects.

## 3. Dental MSC-Based Therapy for Dental Diseases

Considering the multi-lineage potential, dental MSCs are suggested as promising cells for the treatment of dental diseases. Therefore, there have been a variety of therapeutic applications in dental medicine, ranging from preclinical studies (Table 1) to initial clinical trials (Table 2).

### 3.1. Endodontic Diseases

Dental caries and tooth trauma are common diseases associated with the teeth, which destroys the rigid structure of the teeth, both the enamel and dentin, resulting in pulp necrosis and periapical disease. For mature permanent teeth, the current routine clinical treatment is traditional root canal therapy based on pulpectomy, which involves the removal of damaged dental pulp tissue, enlargement of the root canal, and filling of the sterile canal with artificial filling materials [68]. When immature permanent teeth suffer from necrotic pulp/apical periodontitis, tooth development would be arrested, resulting in immature teeth with a thin root dentin and open apices. These teeth need to be treated with special measures based on pulpectomy, including the traditional apexification procedure and an apical mineral trioxide aggregate (MTA) plug [32]. Despite the wide implementation of the current routine treatment, the lack of biological dentin/pulp or dentin-pulp complex and the limitations of existing materials may lead to a great risk of serious reinfection and tooth fracture, thereby resulting in a poor survival rate for teeth. Therefore, the biological regeneration of dentin and pulp could be an ideal and alternative solution to replace defective dental structures in modern dental medicine. Based on different pulp conditions, several novel ideas for dentin-pulp complex and dental pulp regeneration therapy are presented [32]. Firstly, for local regeneration of the dentin-pulp complex following pulpotomy, combining dental MSCs with growth factors or platelet-rich plasma (PRP) is a promising solution to induce DPSCs and capillaries from the residual root pulp tissue and regenerate dentin tissue. Secondly, for a complete regeneration of the dentin-pulp complex for devital tooth after pulpectomy or pulp necrosis, cell homing and cell transplantation are utilized to achieve regeneration of the entire dental pulp for adult permanent teeth or revascularization for immature permanent teeth. However, the traditional revascularization approach fails to regenerate the dentin-pulp complex, unlike novel tissue engineering [68]. With various types of stem cells identified and remarkable breakthroughs in tissue engineering, numerous researches on dental MSC-mediated dentin and dental pulp regeneration have been carried out in animal models and human clinical trials [32].

#### 3.1.1. Dentin Regeneration

The composite construct made up of porcine SHEDs and *β*-TCP scaffold has been directly capped on the created chamber roof defects in the premolars of swine, showing that almost complete dentin regeneration was observed with the newly formed dentin-like structure performing sparse porosity and certain thickness. It is indicated that the novel therapy based on dental MSCs significantly regenerated the dentin-like structure and is useful in direct pulp capping [69]. Subsequent research explored hDPSC-mediated dentin regeneration. hDPSCs were cultured onto the human dentin treated by ethylene diamine tetra-acetic acid and citric acid (hTD) and then implanted in the mouse model. Formation of dentin-like tissues expressing specific dentin markers demonstrated that hDPSCs could be induced by hTD to regenerate the complete dentin tissue *in vivo* [70]. Meanwhile, hDPSCs were seeded onto a novel injectable cell carrier named nanofibrous spongy microspheres (NF-SMS). The result showed that a supported dentin-like tissue was generated in nude mice [71]. However, the narrow root foramen/limited tissue infiltration and blood supply would hold back the application of dental MSCs in clinic. A combination of some powerful growth factors and stem cells could serve as an alternative solution. Zhang *et al.* modified hDPSCs by overexpressing platelet-derived growth factor- (PDGF-) BB, which is a potent mitogenic factor as a mediator in wound healing and tissue repair, and obtained more dentin-like mineralized tissue similar to tooth dentin tissue *in vivo*. Further studies demonstrated that PDGF-BB-modified hDPSCs facilitated stem cell homing via the PI3K/Akt pathway and improved hDPSC-mediated dentin-pulp complex regeneration [72]. Lastly, a novel dentin–pulp-like organoid was developed by constructs mixed with hDPSCs and Matrigel in an odontogenic differentiation medium. The organoid demonstrated a biologically active response to biodentine supplements and suggested hDPSCs as a future approach for tooth regeneration [33]. Although scientific evidence shows a positive trend to dentin regeneration using dental MSCs, especially hDPSCs in animal models, there is a lack of persuasive evidence of clinical trials up to now.

#### 3.1.2. Pulp/Dentin–Pulp-Like Regeneration

Dental pulp plays an indispensable role in maintaining homeostasis of the tooth, but its capacity of self-repair is highly limited. Hopefully, recent preclinical and clinical studies on cell homing and autogenous/allogeneic dental MSCs transplantation have provided further evidence of dental pulp regeneration. Depending on the clinical situation, there are mainly two cell-based pulp-regeneration strategies, partial dental pulp regeneration and whole pulp tissue regeneration [73]. A tentative experiment achieved rat DPSC-mediated partial dental pulp regeneration in rat molars after pulpotomy, suggesting that the remaining healthy pulp tissue could be recoverable and may have the potential to regenerate the lost portion of the dental pulp tissue [74]. Subsequent researches were performed in large animal models to test the feasibility of this regenerative approach. In a beagle dog model, Jia *et al.* transplanted canine DPSCs (cDPSCs) pretreated with simvastatin into immature premolars treated by pulpotomy. Then, regenerated coronal pulp was found filling nearly the entire pulp chamber with newly formed dentin and odontoblastic cells seen in the regenerated area, suggesting that coronal dental pulp regeneration could be realizable by cDPSCs transplantation [75]. Nevertheless, a study implanted pDPSCs/hydrogel into premolars and molars after pulpotomy in a minipig model and only found reparative dentinogenesis without dental pulp regeneration, highlighting the necessity for further investigations to develop a favorable regenerative microenvironment [76].

In recent years, there is growing concern about cell-based regenerative therapy for pulpless teeth. Moreover, several clinical studies are currently underway to confirm the efficacy and safety of stem cell-based regenerative therapy. Inspiring outcomes have been reported for the whole dental pulp regeneration. Nakashima *et al.* developed a composite of drug-approved collagen scaffold and clinical-grade human mobilized DPSCs (MDPSCs) induced by granulocyte colony-stimulating factor (G-CSF) and achieved complete dental pulp regeneration by autologous transplantation with the composite in the mature teeth of dogs after pulpectomy. Similar to the healthy dental pulp tissue, regenerative pulp-like tissue presented good vasculature, innervation, odontoblast-like cells, and recovered function. Moreover, rare adverse effects confirmed the safety of cell therapy for dental pulp regeneration. A notable finding is that there were no significant age-related changes in biological properties and the stability of human MDPSCs *in vitro* and *in vivo* [77]. Then, this team performed a pilot clinical study to further demonstrate the availability and clinical safety of autologous transplantation of MDPSCs in pulpectomized teeth [37]. Functional dentin formation was observed by cone beam computed tomography (CBCT) in three of the five patients. Further study showed that varisble sizes hDPSCs constructs possess the ability of self-organizing and can fill the human tooth root canal to regenerate blood vessel-rich pulp-like tissues after implantation in the subcutaneous space of mice [78]. Much more significantly, whole functional dental pulp tissue regeneration in a minipig was observed after pig DPSC aggregates were implanted into young permanent incisors. And newly formed dental pulp tissue containing an odontoblast layer and blood vessels as well as the expression of neuron markers NeuN indicated that functional dental pulp regeneration could be achieved in a large preclinical animal model [79]. Recently, Xuan *et al.* performed a randomized clinical controlled trial for treating immature permanent teeth injuries due to trauma. Taking apexification as a control group, this study demonstrated that not only could hDPSCs implantation regenerate 3D dental pulp tissue with blood vessels and sensory nerves but it could also show better efficacy and safety of hDPSCs implantation [79]. The majority of MSC-based endodontic treatments were performed in immature permanent teeth of adult patients. Interestingly, a recent case showed a personalized cell therapy in tooth #28 with symptomatic irreversible pulpitis in a 50-year-old man. As reported, hDPSCs were isolated from the inflamed dental pulp tissue of the diseased tooth #28. Combined with leukocyte platelet-rich fibrin (L-PRF) from the patient's blood, expanded hDPSCs were introduced into the prepared root canal. There was a positive response to an electric pulp test and a vitality test after a follow-up period of 36 months, which indicated that this MSC-based therapeutic method contributed to denetal pulp regeneration [80].

### 3.2. Periodontal Diseases

Periodontitis leads to the damage of periodontal tissue including gingiva, cementum, ligament, and alveolar bone [81]. At present, periodontitis is routinely treated by debridement, surgery involving mechanical means, and guided tissue regeneration (GTR), which remain unsatisfactory due to rare regeneration [82]. The ultimate therapeutic goal for periodontal diseases is to regenerate lost periodontal tissues. To address this, cell-based tissue regeneration has become one of the optimal periodontal therapies [81]. Several outstanding reviews have summarized the progress of cell-based regeneration of periodontal tissues [82, 83]. In the current review, we focus on the advance of dental MSC-based therapy for periodontal diseases, particularly periodontitis.

Previous studies of dental MSC-based therapy for periodontal tissue regeneration mainly focus on PDLSCs and DPSCs, but the source of PDLSCs is limited. A recent study discovered that SCAPs could serve as an alternative cell source for periodontitis treatment. Human SCAPs were injected subperiosteally to the surface of bone around the periodontitis defects in minipigs. It demonstrated that local injection of SCAPs improved gingival status and enhanced both bone and cementum regeneration [84]. With the discovery of key factors that maintain the function of SCAPs in periodontal treatment, subsequently, a strategy of gene modification has been studied by Li *et al.* By comparative investigation, they found that SCAPs overexpressing with SFRP2 promoted SCAP-mediated bone, PDL, and cementum regeneration in a minipig periodontitis model [85]. Recently a novel method named cell transfer technology was devised, in which cells were transferred onto a scaffold surface. With this new approach, Iwasaki *et al.* transferred human PDLSCs to the decellularized amniotic membrane (amnion) and transplanted the PDLSC-amnion into a rat with a created dehiscent-type periodontal defect. Newly generated cementum, PDL, and bone were detected, suggesting dental MSC-based treatment as a proposed new technology for periodontal diseases [86]. Conditioned medium generated by dental MSC culture (dental MSC-CM), which contains growth factors, cytokines, and other active substances, is considered as another new trend in periodontal tissue regeneration. Cell-free dental MSC-CM is more convenient and safer to apply in clinic than cell-based therapy. Qiu et al. transplanted collagen membranes loaded with concentrated GMSC-CM and PDLSC-CM into the buccal periodontal defects of molars in rats. More newly formed periodontal tissues were observed in both GMSC-CM and PDLSC-CM [87].

Following supporting evidence provided by numerous animal studies, the first human clinical trial was carried out to treat periodontal osseous defects in three patients through autologous *ex vivo* PDLSCs transplantation [88]. Later researchers devised a novel approach with stem cell assistance in the periodontal tissue regeneration technique (SAI-PRT) bypassing *ex vivo* PDLSCs. In a case report, researchers transplanted the transferable mass consisting of gelatin sponge and soft tissue harboring PDLSCs scraped from cementum and the alveolar socket of the third molar into the intrabony defect of another molar in the same patient. Then, they obtained clinical success with the reduction of probing pocket depth and the recovery of attachment over the evaluation of one year. Although the study is not certain about the number and viability of immediate PDLSCs transplantation, SAI-PRT might be a constructive avenue in the treatment of periodontal osseous defects [89]. Meanwhile, Aimetti *et al.* reported serial cases to explore the clinical potential effects of the application of hDPSCs to treat deep intrabony defects via regenerative therapy. A total of 11 periodontitis patients with intrabony defects received treatment including a minimally invasive flap and autologous hDPSCs loaded on a collagen sponge. Significant clinical improvements and rare adverse effects were observed in a one-year follow-up [90]. Then, the team performed a randomized controlled clinical trial to evaluate the effectiveness of the novel therapeutic strategy as studied above. A remarkable reduction of probing depth (PD), a gain of clinical attachment, and the filling of bone defects in a test group further suggested that this cytotherapeutic approach based on PDLSCs engineering is a safe and innovative strategy to treat severe periodontal defects [91]. Moreover, a case report presented the effect of allogeneic hDPSCs transplantation in periodontal tissue regeneration of an aged periodontitis patient. hDPSCs were obtained from the dental pulp tissue of a 7-year-old donor and expanded. During periodontal surgery of the mesial circumferential bone defect, hDPSCs were seeded into a lyophilized collagen-polyvinylpyrrolidone sponge. After the allogeneic graft, the patient exhibited improved clinical manifestation without any sign of rejection. It is indicated that allogeneic hDPSCs transplantation could induce periodontal tissue regeneration [92].

### 3.3. Therapeutic Strategies in MSC-Based Dental Medicine

**Figure 2:**
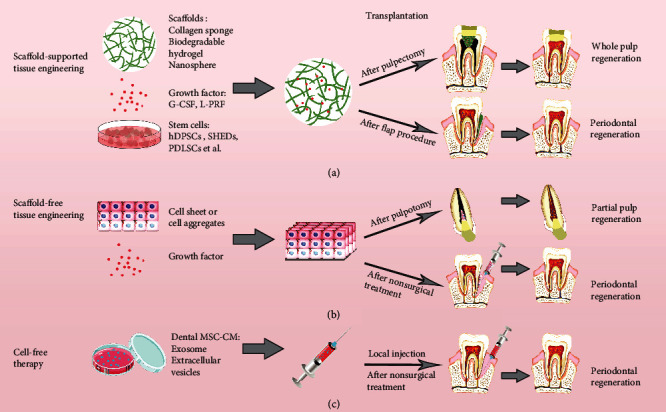
Three therapeutic strategies of endodontic and periodontal diseases using dental MSCs. (a) Dental tissue regeneration through a classic tissue engineering model, consisting of dental MSCs, supporting biomaterial scaffolds, and growth factors. (b) Dental tissue regeneration by tissue engineering without scaffolds. (c) Dental tissue regeneration with a cell-free approach using conditioned medium (CM) with exosomes and/or extracellular vesicles (EVs) secreted by dental MSCs.

Due to their excellent potential for multil-ineage differentiation, dental MSCs are considered as an ideal source for tissue engineering and regenerative dental medicine. To date, researchers are looking for a feasible, safe, and effective approach for regenerative and translational dentistry [16]. Three feasible regenerative strategies based on dental MSCs have been proposed to treat dental diseases in clinic (Figure 2).

#### 3.3.1. Scaffold-Supported Tissue Engineering

Generally, the principles of tissue engineering are based on three elements, including stem cells with multi-lineage differentiation potential, scaffolds as carriers for stem cells, and bioactive molecules inducing differentiation [93]. Dental MSCs are regarded as ideal cells for dental tissue engineering since they possess a shared embryological origin with craniofacial tissue [94]. Biocompatible scaffolds provide a favorable 3D microenvironment for stem cells, which regulate proliferation and differentiation [16]. In regenerative dentistry, current dominating attempts and studies of scaffold-supported tissue engineering include regeneration of dentin, dental pulp, and periodontal tissue and formation of bioroot. In a miniswine model, Zhu *et al*. transplanted autologous and allogeneic swine DPSCs carried by a bioscaffold hydrogel into the root canal space of the miniswine. Orthotopic vascularized pulp-like tissue regeneration was achieved with newly generated dentin-like tissue or osteodentin along the canal walls [95]. Similarly, a study used a root-shaped HA/TCP scaffold with allogeneic swine DPSCs, which was wrapped by a vitamin C-induced allogeneic PDLSC sheet and implanted into the jaw bone socket in swine and successfully regenerated a functional bioroot with a dentinal tubule-like structure and a functional PDL-like structure after six months [96]. Recently, a cell-laden hydrogel encapsulating GMSCs was used to promote craniofacial bone tissue regeneration. This study showed complete bone regeneration around ailing dental implants in rat peri-implantitis [97]. To achieve dentine-pulp complex regeneration, the optimal protocols should integrate cells, biomaterials, and growth factors. However, there are still several issues related to long-term safety and effectiveness, such as host immune rejection, degradation, and potential infection.

#### 3.3.2. Scaffold-Free Strategies for Tissue Engineering

Based on the formation of tridimensional cell-to-cell aggregates without any other external support, scaffold-free technologies avoid the unknown risks of using biomaterials. Two scaffold-free strategies have caught the eye of researchers, which are cell sheets and cell injection. As a unique method of cell processing via culturing in temperature-responsive cell culture dishes or in ascorbic acid, cell sheets have been widely explored and applied in regenerative dentistry [16, 98]. PDLSC cell sheets were autologously transplanted into the denuded root surface in a canine model with a one-wall intrabony defect, and periodontal tissue regeneration was remarkably observed with both cementum and PDL fibers after eight weeks [99]. Cell injection might be a common treatment for periodontal disorders because it is a minimally invasive process. Local injection of allogeneic SCAPs has been shown effective for treating periodontitis by the promotion of periodontal tissue regeneration in a miniature pig model [84]. However, the cell injection approach, independent from the use of any scaffold or biomolecule, has some practical issues, for instance, the risk of losing cell properties in the asepsis storage period and a small application range.

#### 3.3.3. Cell Homing or Cell-Free Therapy

Cell homing is a cell-free approach to repair or regenerate tissue through active recruitment of host endogenous cells to the injured region, mainly via bioactive molecules. Compared with stem cell engraftment, cell homing may evade many hurdles in clinical translation of cell transplantation, including tumorgenicity, antigenicity, host rejection, and infection associated with cell-based therapies [100]. Exosome secreted by dental MSCs could act as paracrine signalers in cell homing. The exosome is one of EVs containing cytokines and microRNAs and plays a vital role in stem cell-based therapy by releasing molecules in target tissues [16]. Furthermore, dental MSCs may provide the secretome/CM with future regenerative therapeutic applications. Compared with the therapy using dental MSCs, dental MSC-CM, which is cell free, exhibits remarkable biological properties, including higher safety, migration activity, and greater ability of odontoblastic differentiation [101]. In a recent work, PDLSC-CM was transplanted into surgically created periodontal defects in a rat, and it was found that PDLSC-CM containing extracellular matrix proteins, enzymes, angiogenic factors, growth factors, and cytokines enhanced periodontal regeneration by suppressing the inflammatory response via TNF-*α* production [102]. The dental MSC-mediated cell-free therapeutic approach is an appealing approach for treating dental diseases and has predominance over cell-based therapy despite some limitations in it. The bioactive molecules involve secretomes released by various populations, and their mechanisms need to be further understood.

## 4. Dental MSC-Based Therapy for Nondental Diseases

### 4.1. Other Oral Diseases

The present therapeutic application of dental MSCs is not limited to endodontic and periodontal diseases. Recently, dental MSC-based therapy for other oral diseases has been proposed in animals and humans, such as craniofacial bone defects, progressive temporomandibular joint (TMJ) arthritis, facial nerve lesions, taste bud loss, and Sjogren's syndrome.

The craniofacial bone defect could be repaired by bone regeneration with dental MSCs [96, 103]. In a well-established rat model with peri-implantitis, GMSC-laden adhesive alginate hydrogels were injected into the bony defect sites around implants, which increased implant survival and the amount of recovered bone [96]. Dental MSCs also hold promise for the treatment of facial nerve injury. Recent experimental evidence showed that a novel method to treat crush injury of rats' facial nerve is via a single application of human SHEDs immediately, which could promote a positive local effect on neuroprotection and remyelination in 2 weeks [104].

A further important application of dental MSCs is for the treatment of TMJ disorders. Common TMJ arthritis often leads to sustained synovitis, cartilage and bone destruction, and pain. Considering the potential immunomodulatory features of human DPSCs, Cui *et al.* tried to locally inject hDPSCs into the articular cavity to treat rat TMJ arthritis. It was found that DPSCs relieved hyperalgesia and synovial inflammation, attenuated cartilage and matrix degradation, and promoted bone regeneration [105]. Dental MSCs have been suggested to promote taste bud regeneration and have promising potential applications in postsurgery tongue reconstruction of patients with tongue cancer [106]. Interestingly, recent work has also suggested that SHEDs exert a protective effect on the secretory function of the salivary gland and exhibit therapeutic potential for the improvement of hyposalivation in Sjogren's syndrome [107].

### 4.2. Extraoral Diseases

Besides widespread application for treating oral diseases, as a powerful autologous stem cell source, dental MSCs also have great therapeutic potential for the treatment of multiple systemic ailments. A recent review has summarized the extensive usage of hDPSCs in the cell-therapeutic paradigm shift to treat various diseases [18]. Other dental MSCs have also been applied in the treatment of extraoral diseases like neurodegenerative diseases and autoimmune and orthopedic disorders. Dental MSCs have a remarkable potential to treat neural diseases such as spinal cord injury (SCI) and peripheral nerve injury, like sciatic nerve and superior laryngeal nerve (SLN) injury, owing to their ability to differentiate into neural-like cells and regenerate neural tissue [107]. SCI is a severe traumatic central nervous system disease resulting in the damage of sensory and motor functions. It has been demonstrated by recent studies that dental MSCs could facilitate functional improvement after SCI in animal models [108–111]. For instance, SHED-CM loaded in collagen hydrogel was injected into the injury site and gained higher Basso, Beattie, and Bresnahan (BBB) scores which suggested that this new cell-free therapeutic approach is conducive to sensory and motor function recovery of SCI [110]. Peripheral nerve injury following traumatic accidents or surgical complications is a severe clinical problem resulting in sensory disturbances, paralysis, and locomotive disability. Because dental MSCs present a great privilege in neurogenic differentiation, they are a hopeful cell source to treat injured peripheral nerves, like the sciatic nerve and SLN [111–113]. For example, the sciatic nerve could be regenerated and repaired after hDPSC implantation or exosome derived from GMSC transplantation in a rat model with sciatic nerve defects [112, 113]. Besides, Tsuruta *et al.* established a novel animal model of SLN injury, which was characterized as having weight loss and drinking behavior changes. The therapeutic effects of systemic administration of SHED-CM in this model showed functional recovery of the SLN and axonal regeneration [114]. Furthermore, hDPSCs have been suggested as an appropriate stem cell source for stroke treatment and acute cerebral ischemia [115, 116].

Also, dental MSCs play an important role in bone and cartilage tissue engineering. Campos *et al.* treated noncritical defects in an ovine model with the biomaterial Bonelike and hDPSCs, and obtained significant radiographic and microscopical evidence of improved bone regeneration [117]. hDPSCs also have been used to treat full-thickness articular cartilage defects. hDPSCs and PRP scaffolds were transplanted into full-thickness cartilage defects in rabbits, resulting in a significant improvement of impaired cartilage and formation of articular cartilage with hyaline-like and fibrocartilaginous tissue [118].

Furthermore, dental MSCs might be another choice for systemic lupus erythmatosus (SLE) therapy and are also effective in reducing a kidney glomerular lesion and perivascular inflammation infiltration [119]. And dental MSCs are able to treat diabetes by obtaining insulin-producing cells or improving diabetic polyneuropathy [119, 120].

## 5. Conclusion

Dental MSCs have been a precious stem cell source in regenerative medicine and have a great therapeutic application potential not only in oral diseases but also in various extraoral diseases. Here, a lot of evidence has demonstrated that dental MSCs are capable of multi-lineage differentiation and are conducive for regenerating and repairing dental tissue. Moreover, some clinical trials with dental MSCs have been completed and demonstrated the efficacy and safety of dental MSC-based therapy for oral diseases. However, these studies are limited, with a limited number of patients and a rather short-term follow-up, so more clinical trials are required before they can be applied effectively and safely in clinic. In addition to a significant potential in dental medicine, dental MSCs have already been considered as an alternative source for nerve and bone regeneration and have therapeutic potential for treating various diseases, such as neural impairment, stoke, bone and cartilage defects, SLE, and diabetes. However, thoroughly understanding the regulatory mechanism of dental MSCs is required before their wide application in clinic.

## Figures and Tables

**Table 1:** Preclinical studies on treatment of dental diseases based on dental MSCs.

Disease target	Type of dental MSCs or their secretions	Animal model	Experiment design			Time	Results or outcome		Reference
			Cell density Administration	Biomaterials/scaffolds	Growth factors		Tissue regeneration	Effect evaluation and safety assessment	
Endodontic diseases	hDPSCs	Nude mice	3 × 10^4^ Subcutaneous transplantation	Human treated dentin (hTD)	—	4, 6, and 8 weeks	Dentin-like tissue	IHE: DSPP, DMP1, and human mitochondria antibodies	[70]
	hDPSCs	Nude mice	2 × 10^6^ Subcutaneous injection 100 *μ*L	Nanofibrous spongy microspheres (NF-SMS)	Odontogenic medium	6 weeks	Dentin-like tissue	IHE: DSPP Safety: NF-SMS almost completely degraded	[71]
	hDPSCs modified by PDGF-BB	Mice	— Subcutaneous transplantation	Porous CPC scaffolds	—	12 weeks	Dentin-pulp complex	Positive markers in IHE: DSPP and PDGF-BB protein secreted endogenous stem/progenitor cell homing	[72]
	Canine DPSCs	Beagle dogs Pulpotomy	1 × 10^7^ Autologous transplantation	Absorbable gelatin sponge	Simvastatin (SIM)	10 weeks	Coronal pulp	RGE: closure of the root apex and thickening of the root canal wall HE: regenerated pulp filling in nearly the entire pulp cavity with odontoblastic cells	[75]
	Porcine DPSCs	Minipigs Pulpectomy	2 × 10^7^ Autologous transplantation Pulp chamber	Injectable nanopeptide hydrogel	—	21 days	Failed No pulp	Micro-CT: reparative mineralized bridge in the residual pulp; failure of partial pulp regeneration	[76]
	Swine DPSCs	Miniswine	2 × 10^7^ Autologous/allogeneic transplantation Root canal	Injectable hyaluronic acid (HyA) gel or collagen TE gel	—	3~5 months	Dentin-like; pulp-like	IHE: DSPP, DMP1, BSP, and Nestin HE: regeneration of vascularized pulp-like tissue with a layer of newly deposited dentin-like tissue or osteodentin along the canal walls. Safety: no overcalcification of the pulp canal space after 5 months of follow-up	[71]
	Canine mobilized DPSCs	Dogs	2 × 10^7^ Autologous transplantation Root canal	Drug-approved collagen	Granulocyte colony-stimulating factor (G-CSF)	14~180 days	Functional dental pulp	HE: regenerative pulp with well vasculature and innervation on day 14 RG: complete obliteration of the enlarged apical portion and lateral and coronal dentin formation Laser Doppler: functional recovery of pulpal blood flow after 90 days Pulp vitality: positive response on day 180 Safety: no adverse effects on both the whole and local	[77]
	Pig DPSCs	Minipig	DPSC aggregates Autologous transplantation Root canals	—	—	3 months	Whole pulp tissue	HE: regenerated pulp tissue containing an odontoblast layer and blood vessels IHE: NeuN	[79]
Periodontal diseases	Human SCAPs	Minipig	2 × 10^6^ Local injection Periodontal defect	—	—	12 weeks	Periodontal tissue	Clinical assessments: PD, GR, and AL loss values decrease CT scan: alveolar bone regeneration HE: remarkable regeneration of periodontal tissues (Sharpey's fibers, periodontal ligament, and cementum)	[84]
	Human PDLSC-CM	Rat	Transplantation Periodontal defect	Collagen sponge	—	4 weeks	Periodontal tissue	Micro-CT: alveolar bone regeneration, decreased exposed root surface area HE: new periodontal tissue formation, dense fibrous connective tissues, periodontal ligament, osteoblast-like cells, small islet-like bone clusters, more united crestal bone	[103]
	SFRP2-human SCAPs	Miniature pig	2 × 10^6^ Local injection Periodontal defect	—	—	12 weeks	Periodontal tissue	Clinical assignment: values of probing depth, gingival recession, and attachment loss Micro-CT: alveolar bone regeneration HE: increased tissue regeneration (increased height of newborn alveolar bone, and mature and thicker new cementum, periodontal ligament, and Sharpey's fibers)	[85]
	Human PDLSCs	Rat	PDLSC-amnion	Amnion	—	4 weeks	Periodontal tissue	Micro-CT images: new bone formation HE: cementum-like, narrow connective tissues, PDL-like tissues	[86]
	Human GMSC-CM	Rat	Transplantation	Collage membrane	—	1, 2, and 4 weeks	Periodontal tissue	HE: newly formed periodontal tissue IHE: TNF-*α*, IL-1*β*, IL-10, BSP-II, and Runx2	[87]

DSPP: dentin sialophosphoprotein; DMP1: dentin matrix protein 1; HE: hematoxylin and eosin; IHE: immunohistochemical stains; PDGF: platelet-derived growth factor; RG: radiographic examination; BSP: bone sialoprotein; DAPI: 4′,6-diamidino-2-phenylindole; PD: probing depth; GR: gingival recession; AL: attachment loss; CM: conditioned medium; SFRPs: secreted frizzled-related proteins; Amnion: decellularized amniotic membrane.

**Table 2:** Current clinical studies on treatment of dental diseases based on dental MSCs.

Application	Source of dental MSCs	Size	Therapeutic strategy			Delivery approach	Follow-up time	Outcome	Reference
			Cells	Scaffolds	Growth factor				
Irreversible pulpitis	Mobilized hDPSCs from 3rd molar	5	Passage 7	—	G-CSF 300 ng	Autologous transplantation	1, 2, 4, 12, 24, 28, and 32 weeks	No adverse events EPT: positive response at 4 weeks MRI: SI in the root canal approached that of the normal pulp in untreated controls after 24 weeks CBCT: lateral dentin formation in three cases at 28 weeks	[37]
Dental trauma with pulp necrosis	hDPSCs from deciduous canine teeth	26	Two hDPSC aggregates containing 1 × 10^8^/ml	—	Extracellular matrix	Autologous implantation	1, 3, 6, 9, 12, and 24 months	No significant side effects after 12 months Digital RVG: continued root development EPT: decrease in sensation thresholds CBCT: apical foramen width decreased, the length of the treated tooth root increased Laser Doppler flowmetry: increase in vascular formation Histology staining: regeneration of 3D whole dental pulp tissue	[79]
Irreversible pulpitis	hDPSCs from inflamed pulp	1	1 × 10^6^/ml	Membrane of collagen	Leukocyte platelet-rich fibrin (L-PRF)	Autologous implantation	6 and 36 months	Remaining free of any symptoms Periapical radiographs: a normal periapical area CBCT: intact periapical bone structures Sensitivity: delayed response to cold EPT: responsive Laser Doppler flowmetry: low blood perfusion	[80]
Periodontal intrabony defects	hDPSCs	11	Micrograft containing cells	Collagen sponge	—	Autologous implantation	6 and 12 months	No adverse events Clinical measurements: PD reduction, CAL increased, pocket closure, gingival improvement Radiographs: intrabony defect decreased	[90]
Periodontal intrabony defects	hDPSCs	15	Micrograft containing cells	Collagen sponge	—	Autologous implantation	6 and 12 months	No adverse events. Clinical measurements: PD reduction, CAL gain Radiographs: bone defect fill	[91]
Periodontal disease	hDPSCs	1	5 × 10^6^/ml	Collagen sponge	—	Allogeneic grafting	3 and 6 months	No signs or symptoms of rejection. Clinical evaluation: tooth mobility, periodontal pocket depth decreased CBCT: bone defect area significantly reduced	[92]

G-CSF: granulocyte colony-stimulating factor; EPT: electric pulp vitality testing; CBCT: cone beam computed tomography; SI: signal intensity; RVG: radiovisiography; PD: probing depth; CAL: clinical attachment level; PPD: probing pocket depth; BOP: bleeding on probing.
